# Intracerebral Hemorrhage with Intraventricular Extension Associated with Loss of Consciousness at Symptom Onset

**DOI:** 10.1007/s12028-020-01180-2

**Published:** 2021-01-21

**Authors:** Jens Witsch, Guido J. Falcone, Audrey C. Leasure, Charles Matouk, Matthias Endres, Lauren Sansing, Daniel Woo, Kevin N. Sheth

**Affiliations:** 1 Department of Neurology, Yale School of Medicine, 15 York Street, LLCI 10th floor, Room 1003C, P.O. Box 208018, New Haven, CT 06520 USA; 2 Department of Neurology, Clinical and Translational Neuroscience Unit, Feil Family Brain and Mind Research Institute, Weill Cornell Medical College, New York, NY USA; 3 Department of Neurosurgery, Yale School of Medicine, New Haven, CT USA; 4 Klinik Und Hochschulambulanz Für Neurologie, Charité-Universitätsmedizin, Berlin, Germany; 5 Department of Neurology and Rehabilitation Medicine, University of Cincinnati, Cincinnati, OH USA

**Keywords:** Intracerebral hemorrhage, Intraventricular hemorrhage, Cohort studies, Patient outcome assessment

## Abstract

**Background:**

In patients with spontaneous intracerebral hemorrhage (ICH), pre-hospital markers of disease severity might be useful to potentially triage patients to undergo early interventions.

**Objective:**

Here, we tested whether loss of consciousness (LOC) at the onset of ICH is associated with intraventricular hemorrhage (IVH) on brain computed tomography (CT).

**Methods:**

Among 3000 ICH cases from ERICH (Ethnic/Racial Variations of Intracerebral Hemorrhage study, NS069763), we included patients with complete ICH/IVH volumetric CT measurements and excluded those with seizures at ICH onset. Trained investigators extracted data from medical charts. Mental status at symptom onset (categorized as alert/oriented, alert/confused, drowsy/somnolent, coma/unresponsive/posturing) and 3-month disability (modified Rankin score, mRS) were assessed through standardized interviews of participants or dedicated proxies. We used logistic regression and mediation analysis to assess relationships between LOC, IVH, and unfavorable outcome (mRS 4–6).

**Results:**

Two thousand seven hundred and twenty-four patients met inclusion criteria. Median admission Glasgow Coma Score was 15 (interquartile range 11–15). 46% had IVH on admission or follow-up CT. Patients with LOC (mental status: coma/unresponsive, *n* = 352) compared to those without LOC (all other mental status, *n* = 2372) were younger (60 vs. 62 years, *p* = 0.005) and had greater IVH frequency (77 vs. 41%, *p* < 0.001), greater peak ICH volumes (28 vs. 11 ml, *p* < 0.001), greater admission systolic blood pressure (200 vs. 184 mmHg, *p* < 0.001), and greater admission serum glucose (158 vs. 127 mg/dl, *p* < 0.001). LOC was independently associated with IVH presence (odds ratio, OR, 2.6, CI 1.9–3.5) and with unfavorable outcome (OR 3.05, CI 1.96–4.75). The association between LOC and outcome was significantly mediated by IVH (beta = 0.24, bootstrapped CI 0.17–0.32).

**Conclusion:**

LOC at ICH onset may be a useful pre-hospital marker to identify patients at risk of having or developing IVH.

## Introduction

Spontaneous intracerebral hemorrhage (ICH) is a devastating neurological disease. One-year mortality is close to 50% [1], and the incidence of ICH has nearly tripled over the past 70 years to most recently 73 cases per 100,000 person-years [2]. New treatment targets and effective therapies are urgently needed.

Intraventricular hemorrhage extension (IVH) of ICH is independently associated with unfavorable outcome [3–5]. Factors that are thought to mediate the worsening effect on outcome are meningitis, induced by blood breakdown products, development of hydrocephalus, and the excess intracranial volume itself leading to increased intracranial pressure [6,7]. An analysis of the INTERACT-2 study cohort showed that IVH was independently associated with both early (within 24 h) and late (up to 7 days) neurological deterioration after ICH [8]. Thus, early recognition of IVH might constitute the first step toward establishing new therapies aiming at containment or removal of intraventricular blood, which might then avert such secondary worsening.

IVH is time-sensitive and associated with hematoma expansion (HE) [4,5,9–11], which in turn can be restricted through blood pressure control, manipulation of coagulation parameters, or surgical intervention [12,13]. In 48%–54% of cases, IVH develops in the first few hours after symptom onset and is already present on hospital admission computed tomography (CT) scan [4,5]. Thus, determination of IVH risk is more likely to impact patient triage, acute management, and potential surgical prearrangements if it happens before arrival at the receiving hospital. Pre-hospital IVH markers may become clinically relevant in the context of mobile stroke units where a CT diagnosis of a developing intracranial hemorrhage can be made before hospital arrival [14]. Alternatively—in the absence of a diagnostic CT scan before hospital arrival—pre-hospital markers may serve as part of diagnostic panels, in conjunction with acute serum biomarkers aiming to distinguish between ischemic and hemorrhagic stroke [15–17].

In patients with spontaneous subarachnoid hemorrhage, loss of consciousness (LOC) at ictus was found to be associated with greater amounts of subarachnoid blood [18]. We speculated that intraventricular extension of ICH might be associated with LOC, too, possibly through a sudden increase in intracranial pressure from blood pouring into the ventricular space rather than being retained by brain tissue. Here, we tested whether LOC at the onset of ICH might serve as an acute clinical marker of IVH on admission or follow-up CT.

## Methods

### Patient Selection and Data Collection

We present a retrospective analysis of the data collected in the Ethnic/Racial Variations of Intracerebral Hemorrhage (ERICH) study. Methods of the ERICH study have been described in detail previously [19]. In brief, ERICH is a multicenter, prospective case–control study that recruited 1000 non-Hispanic Black people, 1000 Hispanics, and 1000 non-Hispanic White people with spontaneous ICH at 41 sites among 19 recruiting centers throughout the USA. In ERICH, ICH is defined as abrupt onset of severe headache, altered level of consciousness, or focal neurological deficit, associated with a focal blood collection within the brain parenchyma on brain imaging. This includes warfarin-associated ICH as well as peripartum ICH. Primary IVH was included in ERICH as well. ICH attributable to malignancies that led to coagulopathy, dural venous sinus thrombosis-associated hemorrhage, vascular malformations, aneurysms, tumors, or hemorrhagic conversion of recent ischemic stroke was excluded. To determine a patient’s eligibility to be included in ERICH, study neurologists reviewed the patient’s clinical presentation and neuroimaging. Clinical characteristics and demographics were prospectively collected through the abstraction of the hospital chart and through interviews with patients and surrogates by trained study investigators. Part of the standardized data collection protocol was the assessment of presenting signs and symptoms (i.e., symptoms that brought the patient in/seek treatment). Mental status at presentation was categorized as “alert and oriented,” “drowsy/somnolent,” “alert but confused,” “coma/unresponsive,” or “posturing.” For the purpose of the present study, patients with a mental status at symptom onset assigned to the categories “coma/unresponsive” or “posturing” were defined as having loss of consciousness at ICH onset.

### Standard Protocol Approvals and Patient Consents

Institutional review boards at each participating institution approved the ERICH study protocol. Written informed consent was obtained from each participant or legally authorized representative. A Spanish-language consent form was available for Spanish only speaking participants or for those who preferred the Spanish consent. Prior to an interview, the patient’s capacity to give consent was screened using a consent comprehension questionnaire. If the patient failed this screen, a legally authorized representative was contacted for enrollment with priority to guardian/power of attorney, spouse, adult children, parents, and siblings, in that order.

### Neuroimaging

Assessment of neuroimaging and determination of hematoma volumes in the ERICH cohort has been described previously [20–22]. Initial and follow-up CT images were used to obtain the following variables: ICH location and ICH and IVH volumes, using semiautomated computerized volumetric analysis (Alice software, Parexel Corporation, Waltham, MA). The presence of IVH was defined as an IVH volume measurement of > 0. In patients with a follow-up CT scan, follow-up ICH volume was determined to assess hematoma expansion (HE). HE was defined as a > 33% or > 6-mL increase in hematoma volume between baseline and first follow-up scan [22]. ICH location was divided in the categories deep (basal ganglia or thalamus), lobar, brainstem, cerebellum, or primary IVH.

### Outcomes

The primary endpoint was the presence of IVH on either admission or follow-up CT. For consistency between admission and follow up imaging, MRI was not considered in the present study. The secondary endpoint was unfavorable outcome 90 days after the index ICH, defined as modified Rankin Scale (mRS) score of 4–6. Health-related quality of life (HRQoL) at 90 days was measured by the EQ-5D (on a scale of− 0.11–1; higher values indicate better HRQoL) and EQ-5D visual analog scale (EQ-5D VAS; on a scale of 0–100; higher values indicate better HRQoL) self-report questionnaires [23,24].

### Statistical Analysis

Analyses were performed using SPSS version 27 (IBM, Armonk, NY, USA). Data are expressed as median and interquartile range (IQR), except for normally distributed data where we used mean and standard deviation (SD). For intergroup differences, we used Mann–Whitney U and *χ*^2^ tests, and binary logistic regression for associations between LOC and 1) IVH on admission CT or follow-up CT and 2) unfavorable outcome at 90 days. In the first binary logistic regression analysis, we included variables based on a combination of statistical significance on univariate analysis and clinical relevance. We included all variables that were significantly different between the two groups (IVH vs. no IVH, Table 2) with the exception of admission Glasgow coma scale (GCS) score (a potential association between admission GCS and IVH was tested in a separate analysis) and any information from prior medication use or past medical history, as these presumably are more difficult to assess accurately in patients with LOC (i.e., to avoid systematic bias). In the second logistic regression analysis, we included the original ICH score components as the most widely accepted variables determining outcome after ICH with the following important modifications: We substituted GCS for LOC and added HE as input variables [4,5,25]. The same analysis was repeated including GCS as covariate with different GCS dichotomization cutoffs.

### Mediation Analysis

We performed a mediation analysis, using a single-mediator model, to determine whether IVH was a variable mediating any relationship between LOC at ICH onset (independent variable) and 3-month outcome (mRS, dependent variable). This method required the use of a continuous mediator variable. We therefore included IVH peak volume rather than IVH as a binary variable [26]. Results were quantified by reporting the mediation coefficients. For the effect of a given variable X on another variable Y, the mediation coefficient demonstrates the change of Y if X changes by one unit. Of note, these coefficients are not proportional to each other, i.e., they cannot be compared directly between different regression analyses. To quantify the effect of LOC on clinical outcome without the mediated effect through IVH, we calculated the variation quotient (C–C′/C, with C being the total effect and C′ being the IVH-mediated effect of LOC on outcome).

For this analysis, we used the SPSS macro “process” version 3.5. In the mediation analysis, 95% confidence intervals (CI) were bootstrapped, generated based on 5000 repetitions. The threshold of statistical significance was set at alpha = 0.05.

## Results

Among 3000 ICH cases enrolled in the ERICH study, we excluded 144 cases because of missing CT volumetric measurements and 134 cases because they had a seizure at symptom onset. Baseline characteristics of included patients (*n* = 2724) are shown in Table 1. Those with LOC (*n* = 352) compared to those without LOC (*n* = 2372) were younger (mean 60 vs. 62 years, *p* = 0.005) and had a greater proportion of women among them (49 vs. 40%, *p* = 0.04). Patients with initial LOC had a lower median GCS score upon ED arrival (median 6 vs. 15, *p* < 0.001), greater admission systolic blood pressure (200 vs. 184 mmHg, *p* < 0.001), and serum glucose (158 vs. 127 mg/dl, *p* < 0.001). Two hundred and seventy-one (77%) patients with LOC had IVH on either admission or follow-up CT scan compared to 978 (41%) patients without LOC (*p* < 0.001). Both initial ICH volume (26 vs. 10 ml, *p* < 0.001) and peak ICH volume (28 vs. 11 ml, *p* < 0.001) were significantly greater among patients with LOC compared to those without LOC. Characteristics of patients with the presence of IVH on either admission or follow-up CT scan compared to those without IVH on any CT scan are shown in Table 2. Table 3 summarizes hospital interventions, in-hospital mortality, 3-month functional (mRS), and quality of life outcomes (EQ5D) of patients stratified by the presence or absence of LOC at ICH onset. In-hospital mortality in patients with LOC was 38% compared to 7% in those without LOC (*p* < 0.001). 27% with LOC had unfavorable functional outcome at 3 months, compared to 23% in patients without initial LOC (*p* < 0.001). EQ5D at 3 months indicated significantly worse quality of life in patients with initial LOC in most categories, with the exceptions of pain, anxiety, and depression, which were similar between both groups (*p* = 0.09 in both categories).

**Table 1:** Baseline characteristics of patients, stratified by initial loss of consciousness

Characteristic^a^	LOC present (*n* = 352)	LOC absent (*n* = 2372)	*p * value
Age, mean (SD), y	60 (15)	62 (14)	0.005
Female	171 (49)	958 (40)	0.04
Race			0.3
White	215 (61)	1539 (65)	
Black	137 (39)	829 (35)	
Modified Rankin before ICH	0 (0–1)	0 (0–1)	0.5
Medical history			
Diabetes	103 (29)	663 (28)	0.3
Hypertension	281 (80)	1917 (81)	0.8
Elevated cholesterol	90 (26)	760 (32)	0.07
CAD	34 (10)	318 (13)	0.1
AF	25 (7)	230 (10)	0.2
TIA	10 (3)	115 (5)	0.1
CHF	22 (6)	175 (7)	0.6
Home medications			
Aspirin	57 (16)	651 (27)	< 0.001
Non-aspirin antiplatelet	20 (6)	135 (6)	1.0
Warfarin	26 (7)	216 (9)	0.3
Statin	74 (21)	592 (25)	0.1
Clinical			
Glucose value by EMS	146 (116–198)	125 (106–154)	< 0.001
ED Glasgow Coma Score	6 (3–9)	15 (13–15)	< 0.001
ED SBP	200 (167–226)	184 (158–213)	< 0.001
Laboratory testing in emergency department			
Serum glucose	158 (130–201)	127 (106–163)	< 0.001
Hemoglobin	13.6 (12.2–15.1)	13.8 (12.6–15.0)	0.2
Platelet count	231 (182–272)	219 (177–266)	0.06
PTT	27 (25–30)	29 (26–32)	< 0.001
First INR	1.0 (0.98–1.10)	1.0 (1.0–1.1)	0.7
CT characteristics			
Lobar hemorrhage location	87 (25)	731 (31)	0.02
Initial CT ICH volume	26 (10–58)	10 (4–24)	< 0.001
Peak ICH volume	28 (12–64)	11 (4–27)	< 0.001
ICH expansion (> 33%)	36 (16)	275 (17)	0.7
IVH on any CT scan	271 (77)	978 (41)	< 0.001
Peak IVH volume	8 (0.3–32)	0 (0–4)	< 0.001

*AF* atrial fibrillation, *CAD* coronary artery disease, *CHF* congestive heart failure, *CT* computed tomography, *ED* emergency department, *EMS* emergency medical services, *ICH* intracerebral hemorrhage, *INR* international normalized ratio, *IVH* intraventricular hemorrhage, *LOC* loss of consciousness, *PTT* partial thromboplastin time, *SBP* systolic blood pressure, *SD* Standard deviation, *TIA* transient ischemic attack, *y* year

^a^Data are represented as number (%) or median (interquartile range), unless otherwise specified

**Table 2:** Baseline characteristics of patients, stratified by the presence versus absence of IVH on any hospital CT scan

Characteristic^a^	IVH present (*n* = 1249)	IVH absent (*n* = 1475)	*p* value
Age, mean (SD), y	62 (14)	62 (14)	1.0
Female	520 (42)	609 (41)	0.9
Race			0.048
White	785 (63)	969 (66)	
Black	464 (37)	502 (34)	
Modified Rankin score before ICH	0 (0–1)	0 (0–1)	0.3
Medical history			
Diabetes	347 (28)	419 (28)	1.0
Hypertension	1019 (82)	1179 (80)	0.06
Elevated cholesterol	374 (30)	476 (32)	0.5
CAD	162 (13)	190 (13)	0.8
AF	119 (10)	136 (9)	0.6
TIA	60 (5)	65 (4)	0.5
CHF	93 (7)	104 (7)	0.6
Home medications			
Aspirin	320 (26)	388 (26)	0.7
Non-aspirin antiplatelet	75 (6)	80 (5)	0.5
Statin	317 (25)	349 (24)	0.3
Warfarin	112 (9)	130 (9)	0.9
Clinical			
LOC at ICH onset	271 (22)	81 (6)	< 0.001
Glucose value by EMS	130 (107–165)	125 (106–156)	0.2
ED Glasgow Coma Score	13 (8–15)	15 (14–15)	< 0.001
ED SBP	190 (163–218)	180 (155–212)	< 0.001
ED DBP	104 (87–122)	99 (83–118)	< 0.001
Laboratory testing in emergency department			
Serum glucose	140 (116–177)	122 (104–161)	< 0.001
Hemoglobin	13.8 (12.5–15.0)	13.8 (12.6–15.0)	0.9
Platelet count	221 (177–270)	220 (177–263)	0.4
PTT	28 (25–31)	29 (26–32)	< 0.001
First INR	1.0 (1.0–1.1)	1.0 (1.0–1.1)	0.6
CT characteristics			
Initial CT ICH volume	15 (6–39)	8 (3–20)	< 0.001
Peak ICH volume	18 (8–43)	9 (3–23)	< 0.001
ICH expansion (> 33%)	155 (12)	156 (11)	0.9
Lobar hemorrhage location	303 (24)	515 (35)	< 0.001
3-month modified Rankin score	3 (2–4)	2 (1–3)	< 0.001

*AF* atrial fibrillation, *CAD* coronary artery disease, *CHF* congestive heart failure, *CT* computed tomography, *DBP* diastolic blood pressure, *ED* emergency department, *EMS* emergency medical services, *ICH* intracerebral hemorrhage, *INR* international normalized ratio, *IVH* intraventricular hemorrhage, *LOC* loss of consciousness, *PTT* partial thromboplastin time, *SBP* systolic blood pressure, *SD* Standard deviation, *TIA* transient ischemic attack, *y* year

^a^Data are presented as number (%) unless otherwise specified

**Table 3:** Hospital interventions, withdrawal of care and outcomes between patients with and without LOC

Hospital interventions	LOC present (*n* = 352)	LOC absent (*n* = 2372)	*p* value
Craniotomy for clot evacuation	44 (13)	170 (7)	0.19
ICP monitoring	154 (44)	343 (15)	< 0.001
Intraventricular drain placed	143 (41)	351 (15)	< 0.001
Shunt placement	34 (10)	97 (4)	< 0.001
Intraventricular tPA	19 (5)	31 (8)	0.01
Clinical outcome			
In-hospital mortality	132 (38)	163 (7)	< 0.001
Comfort measures only (CMO)	97 (28)	187 (8)	< 0.001
3-month mRS score	4 (3–5)	3 (1–4)	< 0.001
3-month unfavorable mRS (4–6)	95 (27)	537 (23)	< 0.001
3-month Barthel score	50 (10–90)	90 (55–100)	< 0.001
EQ5D mobility	2 (2–3)	2 (1–2)	< 0.001
EQ5D self-care	2 (2–3)	2 (1–2)	< 0.001
EQ5D usual activities	2 (2–3)	2 (1–3)	< 0.001
EQ5D pain	2 (1–2)	2 (1–2)	0.09
EQ5D anxiety/depression	2 (1–2)	1 (1–2)	0.09
EQ5D health state score	60 (44–70)	70 (50–80)	< 0.001

*ICP* intracranial pressure, *LOC* loss of consciousness, *mRS* modified Rankin score, *tPA* tissue plasminogen activator

### LOC and IVH

In order to determine whether LOC at ICH onset was independently associated with the presence of IVH on any in hospital CT scan, we conducted a multivariate regression analysis controlling for clinical variables differing between patients with and without IVH (Table 2). LOC at ICH onset was independently associated with IVH occurrence (OR 2.6, CI 1.9–3.5) (Table 4). Exclusion of patients whose index ICH was located in the brainstem did not significantly alter this association (OR 2.8, CI 2.0–3.9), neither did exclusion of patients with positive drug screen on admission (OR 2.6, CI 1.6–3.9). We repeated this regression analysis substituting LOC at symptom onset with GCS < 8 upon ED arrival, as assessed by ED staff showing a similarly strong association between GCS and IVH (OR 2.6, CI 1.9–3.5).

**Table 4:** Binary logistic regression analysis of the presence of IVH on any CT scan

	OR	95%CI	*p* value
First systolic BP in ED [mmHg]	1.001	0.99–1.002	0.46
Serum glucose upon ED admission	1.001	1.0–1.003	0.067
Partial thromboplastin time upon ED admission	0.99	0.98–1.00	0.052
Lobar ICH location	0.30	0.23–0.38	< 0.001
Peak ICH volume	1.03	1.02–1.034	< 0.001
Loss of consciousness at ICH onset	2.6	1.90–3.54	< 0.001

*BP* blood pressure, *CI* confidence interval, *CT* computed tomography, *ED* emergency department, *IVH* intraventricular hemorrhage, *OR* odds ratio

### LOC and Unfavorable Outcome at 90 days

LOC at ICH onset was independently associated with unfavorable outcome at 90 days (OR 3.05, CI 1.96–4.75), controlling for HE and the components of the original ICH score, but substituting GCS on ED admission for LOC (Table 5). Again, exclusion of brainstem ICH cases did not significantly alter this association (OR 3.34, CI 2.16–5.14), neither did exclusion of patients with positive drug screen (OR 3.9, CI 2.4–6.3). When adding GCS to the regression analysis, depending on the GCS dichotomization cutoff, LOC was or was not associated with 90-day unfavorable outcome (Table 6).

**Table 5:** Binary logistic regression analysis of unfavorable outcome at 3 months (mRS 4–6). ICH score elements, hematoma expansion, and initial loss of consciousness as covariates

	OR	95%CI	*p* value
Age > 80 years	3.5	2.333–5.154	< 0.001
Initial ICH volume > 30 ml	2.9	2.11–4.072	< 0.001
Hematoma expansion*	2.4	1.728–3.324	< 0.001
Infratentorial hemorrhage location	1.03	0.688–1.545	0.88
Loss of consciousness at ICH onset	3.05	1.959–4.745	< 0.001

*CI* confidence interval, *ICH* intracerebral hemorrhage, *OR* odds ratio

*HE defined as a > 33% or > 6-mL increase in hematoma volume between admission and follow-up CT

**Table 6:** Binary logistic regression analysis of unfavorable outcome at 3 months (mRS 4–6) controlling for ICH score elements and hematoma expansion (Table 5) as covariates and adding various GCS dichotomizations

	OR	95%CI	*p* value
GCS less than 15	2.8	2.166–3.650	< 0.001
Loss of consciousness at ICH onset	2.1	1.302–3.244	0.002
GCS less than 13	3.7	2.685–5.097	< 0.001
Loss of consciousness at ICH onset	1.5	0.880–2.387	0.145
GCS less than 8	2.1	1.252–3.430	0.005
Loss of consciousness at ICH onset	2.2	1.331–3.600	0.002
GCS less than 6	1.5	0.765–2.822	0.248
Loss of consciousness at ICH onset	2.7	1.692–4.404	< 0.001
GCS 3	1.9	0.861–4.314	0.11
Loss of consciousness at ICH onset	2.7	1.695–4.297	< 0.001

*CI* confidence interval, *GCS* Glasgow coma scale, *ICH* intracerebral hemorrhage, *OR* odds ratio

The combination of discontinuation of aggressive medical care and do-not-resuscitate orders was placed in 9.9% of study participants. Excluding these patients from the regression analysis, as shown in Table 5, did not substantially change the association between LOC and 90-day outcome (OR 3.05, CI 1.96–4.76, analysis not shown).

### Mediation Analysis

To test whether IVH was mediating a portion of the effect of LOC at ICH onset on outcome at 90 days, we regressed LOC and IVH onto unfavorable mRS at 90 days (dichotomized) together. The regression model revealed positive and statistically significant direct effects from LOC to IVH (beta = 11.62, standard error = 1.04, *p* < 0.001), IVH to unfavorable outcome (beta = 0.02, standard error = 0.003, *p* < 0.001), and LOC to unfavorable outcome (beta = 0.72, standard error = 0.12, *p* < 0.001). The indirect effect of LOC to unfavorable outcome, mediated by IVH, was statistically significant (beta = 0.24, bootstrapped standard error 0.04, bootstrapped CI 0.17–0.32). The variation quotient (C–C′/C) was 67%. The results were similar in a sensitivity analysis in which we excluded patients whose index ICH was located in the brainstem. In this analysis, the indirect effect of LOC to unfavorable outcome, mediated by IVH, was similar to the one in the primary analysis (beta = 0.27, bootstrapped standard error 0.04, bootstrapped CI 0.19–0.36, variation quotient 58%).

## Discussion

Among ICH cases from the multicenter case–control study ERICH, we found that loss of consciousness at the onset of ICH was independently associated with the presence of IVH on either admission or follow-up CT. LOC was also associated with 90-day unfavorable outcome when adjusting for components of the ICH score and HE. These findings held up in sensitivity analyses excluding patients with positive drug screening on hospital admission and those whose ICH was located in the brainstem. When controlling for GCS upon ED admission, the association between LOC and outcome was partially preserved depending on which GCS cutoff was used, suggesting overlapping prognostic information between LOC and admission GCS. A mediation analysis revealed that the association between LOC and outcome was significantly mediated through IVH. Because brainstem bleeds are in close proximity to the reticular activating system and the ventricular system, we conducted a sensitivity mediation analysis in which we excluded patients with brainstem bleeds. This analysis produced similar results as the primary analysis suggesting that the mediated effect of LOC on outcome through IVH was not driven or significantly attenuated by hematoma location in the brainstem. Overall, these findings suggest that in patients with ICH, LOC at symptom onset is a marker of IVH which in turn worsens outcome.

Prior studies have established that intraventricular extension of ICH is a time-dependent process which can occur with delay after a primarily parenchymal bleed and that in 7%–10% of all ICH cases IVH extension happens after the hospital admission CT scan [4,5,9]. With regard to its effect on outcome, IVH is not an “all or nothing” event. Increasing degrees of IVH correlate with an increasing risk of worse outcome [27–30]. These findings suggest that there might be a subgroup of patients with ICH in whom ICH is diagnosed early enough to potentially prevent IVH or contain an early stage IVH through blood pressure control or surgical intervention [12,31]. Certainly, such interventions would still need to be tested in prospective trials with regard to their efficacy. Despite the established relationship between IVH and clinical outcome, dedicated studies focusing on prediction of IVH were not available so far. Associations between greater ICH volume and hyperglycemia upon hospital arrival on the one hand and occurrence of IVH on the other have been reported. An inverse relationship between lobar hemorrhage location and IVH has been shown as well [4,5,9,32]. We reproduced the inverse relationship between lobar location of the ICH and IVH in our study. However, systolic BP and admission serum glucose were not associated with the presence of IVH in multivariate analysis (Table 4). Of note, several of the baseline characteristics of patients with LOC differed from those without LOC. Patients without LOC were older, more frequently male, more often had hyperlipidemia, more frequently were taking aspirin as home medication, and had greater PTT values on admission. Patients without IVH also had a greater median admission PTT. These differences might be driven by a greater prevalence of lobar ICH location in those without LOC (compared to those with LOC) and in those without IVH (compared to those with IVH). Patients with lobar ICH may have included a greater proportion of patients with amyloid angiopathy, a demographic that is typically older and has more comorbidities.

The novel finding our study adds is the identification of LOC as an acute clinical marker of IVH. LOC had the strongest independent association with IVH, and—being an early data point, collected before hospital arrival—LOC might be clinically more meaningful for early IVH prediction than the GCS score in the emergency department, or CT and laboratory findings which typically do not become available until hospital arrival. Our head-to-head comparison of the pre-hospital LOC variable and the dichotomized GCS in the ED shows that LOC overpowers the GCS at lower cutoff values (less than 8) that tend to align more with “loss of consciousness.” This suggests that the early level of consciousness after ICH is more meaningful for outcome prognostication than the assessment of consciousness in the ED, possibly because the pre-hospital level of consciousness is less likely confounded by sedating medications.

In our regression analysis (Table 4), we controlled for ICH volume and thus established that IVH is not simply a surrogate marker of a large parenchymal hematoma more likely to cause IVH and coma at symptom onset through mass effect [5]. However, we were not able to control for midline shift, hydrocephalus, or sudden increase in intracranial pressure (data not available), conditions that may co-occur with IVH and then lead to compression of brain structures associated with maintaining consciousness. We controlled for ICH location, but a greater anatomical resolution than large cohort studies typically provide would be needed to assess for hematoma locations as a primary cause for LOC. Studies investigating coma in patients with ICH have, among others, implicated brain structures with the presence of coma that are close to the ventricular system, for example lesions of the head of the caudate or the paramedian tegmentum [33,34]. In accordance with these findings from prior studies, patients with LOC had a greater proportion of deep and a smaller proportion of lobar hemorrhages in our study (*p* = 0.02). Thus, proximity of the hematoma to the ventricles may have influenced the association between impaired consciousness and IVH extension. Lastly, one may speculate that IVH may be an initial event with a cascade of downstream effects leading to LOC rather than being the direct cause of LOC. For example, IVH may cause hydrocephalus, which in turn may lead to ICP increase, which then might interfere with either cortical function or lead to different degrees and types of herniation. All of these mechanisms are well-described causes of coma [35,36].

Although our article reports associations between clinical variables, which are relevant for diagnosis rather than therapy of IVH, it implicitly raises the question what the optimal intervention is in response to IVH detection. The literature reports conflicting findings about whether removal of blood and blood breakdown products or reversal of hydrocephalus should be the primary goal of surgical intervention [37–39]. The question is difficult to address in research studies as insertion of an external ventricular drain will typically treat both aspects of the pathology. The results of the CLEAR III trial suggest that intraventricular clot removal through insertion of an external ventricular drain in combination with alteplase irrigation does not generally improve clinical outcome in all patients with ICH. However, it may be beneficial in selected subgroups or in combination with a lumbar drain [40–42]. More research is needed to define patient subgroups and interventions that target IVH.

The results of our study should be seen as preliminary and need confirmation in future prospective trials. If the association between LOC at ICH onset and IVH can be reproduced, its use in conjunction with other pre-hospital tests might allow efficient triage. Possibly, this might lead to earlier EVD insertion in those who need it.

Our study has limitations. First, although the study data were collected prospectively, our analysis was retrospective, which has conceptual limitations. We cannot exclude a degree of inaccuracy regarding assessment and documentation of the initial symptom (loss of consciousness); however, data in ERICH were collected carefully by dedicated study investigators. Second, the etiology and dynamics of the initial LOC were not addressed in this study. Importantly, data on the presence of hydrocephalus on CT were not available. Hyperacute onset hydrocephalus may contribute to the association between LOC and IVH, and it certainly contributes to the association between IVH and clinical outcome [37,39]. Many different trajectories after initial LOC are possible, ranging from recovery to progression to brain death. Future studies will hopefully clarify these different trajectories as well as how initial LOC relates to early and delayed neurological deterioration. Third, we did not differentiate between patients with initial and delayed IVH in our analysis. The distinction between the two depends on the time interval between symptom onset and imaging [5], a variable that was not available with hour/minute resolution for all cases in ERICH. To account for that, we decided to analyze patients with initial and delayed IVH together, and to use, wherever available, peak ICH volume instead of ICH volume on admission CT. Peak ICH volume can be assumed to be a more robust measure in the context of variable symptom-to-imaging time intervals. Of note, around 30% of ERICH cases did not have follow-up CT imaging. Thus, in these cases only one ICH volume measurement is available which precludes assessment of HE. Dedicated studies would be needed to assess whether LOC or decline of mental status after hospital arrival is associated with delayed IVH.

Fourth, there are limitations inherent to mediation analysis as a statistical technique; mediation analysis may help to identify statistical associations, but it cannot prove causality between mediator and dependent variable. Fifth, this is a clinical study and correlations between clinical and imaging findings cannot be made with high accuracy. Small prospective studies with high-resolution imaging technology might be more appropriate study designs for this purpose. Lastly, the ERICH study includes only people of White or Black race (both non-Hispanic) or Hispanic ethnicity which may limit the generalizability to other populations.

## Conclusions

In this analysis of ICH cases from the prospective multicenter ERICH cohort, pre-hospital LOC was strongly associated with the presence of IVH on admission or follow-up CT brain scan. This information may become clinically useful for patient triage and risk stratification in future trials. LOC was also associated with long-term functional outcome after ICH, but a significant portion of this effect was mediated through IVH. The findings of this report emphasize the importance of IVH as a treatment target in patients with ICH. Future studies are needed to characterize how LOC can be integrated into clinical tools that predict IVH, and how IVH and LOC may connect mechanistically.
